# Dementia Care and Well-Being: Dyadic and Relational Considerations

**DOI:** 10.1093/geroni/igaf122.2072

**Published:** 2025-12-31

**Authors:** Hanamori Skoblow, Megan Gilligan, Christine Proulx

## Abstract

Caregiving in the context of dementia exerts profound impacts on personal well-being, for both the person living with dementia and their care partners. Dementia care is inherently relational as care is often provided by a variety of care partners, most of whom are unpaid family members and nonrelatives. The well-being of people with dementia and their care partners are also interconnected, emphasizing the need to understand the broader social context in which caregiving takes place. This symposium brings together four studies that explore how dementia care shapes well-being among family caregivers and people living with dementia. This research highlights a diverse range of family relationships and relational processes, including siblings navigating parent care together, couples managing early-stage Alzheimer’s disease, adult daughters caring for a parent with dementia, and caregivers raising children while caring for an older adult. Dr. Skoblow will examine the implications of discrepancies in siblings’ views of their parents’ dementia symptoms on caregiver burden. Dr. Bonds Johnson will investigate life course factors that are associated with quality of life in both members of African American parent-adult daughter dementia dyads. Ms. Lee will consider how dementia knowledge, empathy, and marital quality relate to incongruence in romantic partners’ perceptions of care needs. Dr. Nemmers will look at psychological well-being in sandwiched caregivers and the older adults receiving care, highlighting differences by dementia status. The discussant, Dr. Proulx, will contextualize these findings within the broader literature on family caregiving, cognitive impairment, and dyadic research.

